# Impacts of a delayed and slow-paced vaccination on cases and deaths during the COVID-19 pandemic: a modelling study

**DOI:** 10.1098/rsif.2022.0275

**Published:** 2022-05-25

**Authors:** Gustavo Barbosa Libotte, Lucas dos Anjos, Regina Célia Cerqueira de Almeida, Sandra Mara Cardoso Malta, Roberto de Andrade Medronho

**Affiliations:** ^1^ National Laboratory for Scientific Computing, Petrópolis, Rio de Janeiro, 25651-075 Brazil; ^2^ Institute of Collective Health, School of Medicine, Federal University of Rio de Janeiro, Rio de Janeiro, 21941-598 Brazil

**Keywords:** COVID-19, low vaccination coverage, delayed start of vaccination, resurgence of cases, avertable deaths

## Abstract

In Brazil, vaccination has always cut across party political and ideological lines, which has delayed its start and brought the whole process into disrepute. Such divergences put the immunization of the population in the background and create additional hurdles beyond the pandemic, mistrust and scepticism over vaccines. We conduct a mathematical modelling study to analyse the impacts of late vaccination along with slowly increasing coverage, as well as how harmful it would be if part of the population refused to get vaccinated or missed the second dose. We analyse data from confirmed cases, deaths and vaccination in the state of Rio de Janeiro in the period between 10 March 2020 and 27 October 2021. We estimate that if the start of vaccination had been 30 days earlier, combined with efforts to drive vaccination rates up, about 31 657 deaths could have been avoided. In addition, the slow pace of vaccination and the low demand for the second dose could cause a resurgence of cases as early as 2022. Even when reaching the expected vaccination coverage for the first dose, it is still challenging to increase adherence to the second dose and maintain a high vaccination rate to avoid new outbreaks.

## 1. Introduction

As of 25 February 2020, when the first case of infection with SARS-CoV-2 (severe acute respiratory syndrome coronavirus 2) was reported in Brazil, the country has accumulated more than 21.8 million confirmed cases and, on 17 November 2021, Brazil’s death toll topped 610 000. To date, nearly 9% of all cases in the world were identified in Brazil and, considering a 7-day rolling average, the country has had at least a thousand deaths per day for more than 240 days since the onset of the epidemic. SARS-CoV-2 circulated undetected in Brazil for more than a month [1] and, even after Brazil declared COVID-19 (coronavirus disease) a national public health emergency on 3 February 2020 [2], the Brazilian government has managed the epidemic very loosely so far [3–5], without a cooperative effort and strategic planning to fight the pandemic. Brazil also faces many economic and socio-cultural challenges that affect mitigation strategies, such as a large disparity in the mortality rate in economically disadvantaged regions [6], the uneven geographic distribution of intensive care unit (ICU) beds [7,8] and lack of investment and vulnerability of the health system [9]. Each federal unit is self-governing for decisions regarding efforts to curb the spread of the disease [10], which leads to inequalities, such as unbalanced social distancing measures and lack of mass testing and viral spread tracking.

The long-awaited roll-out of vaccination programmes against COVID-19 from across the globe has fuelled hope for a reduction in the incidence of cases and deaths, as well as the resumption of economic and social activities. Despite the critical situation in containing the ensuing epidemic and the resurgence of cases (especially with the emergence of new variants), compared to other countries Brazil had delays in starting the vaccination campaign [11], which began on 17 January 2021. Even with a slight increase in the pace of vaccination in recent months, vaccination efforts remain far below what is required, with only 72.62% of the national population having received at least one dose by 6 November 2021. In turn, the second dose began to be administered on 5th February, and since then only 56.65% of the population has been immunized. To achieve full coverage for people aged 18 and over by the end of 2021, Brazil needed an average of 1.5 million doses of vaccine administered per day [12]. As of 6 November 2021, the average is approximately 946 000 per day (first dose and second dose or single dose). Currently, the population benefits from vaccines from Pfizer-BioNTech, Oxford-AstraZeneca, Janssen and Sinovac (the latter two approved for emergency use up to the time of writing this paper).

The epidemiological situation in some states is particularly worrisome due to the level of government intervention, investments in health, the pace of vaccination and population mobility [13–15]. Political polarization and the spread of fake news also hamper the fight against COVID-19 and the adoption of non-pharmaceutical interventions (NPI) [16,17]. Rio de Janeiro is one of the most important states in Brazil (hereinafter referred to as Rio de Janeiro), in terms of demographic density and economic relevance. With an estimated population of approximately 17.3 million inhabitants in 2020, the state of Rio de Janeiro is more populous than countries like Belgium, Portugal and Sweden. The first case reported in the state was that of a traveller returning from Italy and, since then, Rio de Janeiro has been one of the states in which the epidemic has grown the fastest, reaching a rate of contagion (in terms of the basic reproduction number) between 2.2 and 4.9 [13]. The progress of vaccination in the state follows the slow pace of the rest of the country: 12.76 million people had received the first dose (73.77% of the population) and 8.96 million people had received the second dose (51.78% of the population) as of 6 November 2021. On average, approximately only 74 800 vaccines have been administered per day since the start of the vaccination campaign in the state, on 20 January 2021.

Although Brazilians’ tendency towards vaccination compliance is relatively high [18], some factors were partly responsible for the slowness of the mass vaccination campaign. The country is paying a price for the slow pursuit of vaccines early on, especially regarding the federal government’s rejection of vaccines from Pfizer in mid-2020 [12], in addition to the revoking of the agreement signed with Sinovac [19]. Millions of people are also missing their second dose—especially because of misinformation, assuming that just one dose provides the expected immunity [20,21]—and also owing to temporary interruptions of vaccination services, a lack of shots, logistical problems or the absence of supplies (particularly active pharmaceutical ingredient) [22,23]. Furthermore, there are on the one hand people who try to jump the queue to get vaccinated early, and on the other hand those who choose not to get vaccinated, seemingly motivated by political ideology [24].

Therefore, it is essential to investigate the likely consequences of such events and circumstances regarding the burden of the epidemic. For this purpose, this study aims to at investigate the following issues:

— What would be the influence of bringing forward or delaying the vaccination roll-out?
— How effective would a faster vaccination process be in mitigating the epidemic?
— How many deaths could have been averted if there had been more efforts to obtain and manage vaccines?
— How harmful is the choice of part of the population not getting vaccinated?
— What is the effect of not taking the second dose of the vaccine on the population?

In this context, the objective of this work is to provide an analysis of scenarios related to the epidemic in Rio de Janeiro, one of the most important states in Brazil in terms of demographic density and economic relevance, to answer the issues raised by employing computational simulations whose results can be compared to the current situation of the epidemic in the state. The general framework we propose can be extended to analyse the situation of the epidemic in any region.

## 2. Methods

### 2.1. Model description

We extend the well-known SIR (susceptible–infected–removed) model [25], aiming to incorporate the effects of vaccination in the population. Initially, assume that *β*(*t*) is the transmission rate over time and *γ* is the removal rate. The gain in the infective class (*I*) is at a rate proportional to the product of the contact rates and transmission probability between infectives and susceptibles (*S*); that is, the rate of new incidences is given by *β*(*t*)*S*(*t*)*I*(*t*)/*N*, where *N* is the population size. In turn, the rate at which infected individuals move into the removed class (*R*) is given by *γI*(*t*). Of note, we also compute the number of dead individuals, once infected, which are eventually moved into the dead class (*D*) at a rate of *ρI*(*t*), where *ρ* is the death rate.

Assume that both susceptible and infected individuals can be vaccinated (the latter are able to be vaccinated as they may be asymptomatic). Considering that *n* different vaccines can be administered in a population, individuals vaccinated with a given vaccine *i* are moved into the corresponding vaccinated class (*V*_*i*_) at a rate equal to *ν*_*i*_(*S*(*t*) + *I*(*t*)), where *ν*_*i*_ is the vaccination rate associated with vaccine *i*, for i=1, …, n. Individuals remain in compartment *V*_*i*_ for the period equivalent to the interval between doses (when applicable), which is given by 1/*τ*_*i*_. After this period, vaccinated individuals are considered immune and therefore moved into the removed class, taking into account the efficacy of the corresponding vaccine, *η*_*i*_. If immunity is not acquired with proper vaccination, vaccinated individuals may become susceptible again, whose class is fed back proportionally to (1 − *η*_*i*_)*V*_*i*_.

The model also covers two other aspects inherent to the vaccination process: first, part of the population eligible to be vaccinated can choose not to take both doses of the vaccine (when applicable). In terms of vaccine efficacy, such individuals have only partial protection, which we denote by ${\bar{\eta }}_{i}$, an impaired efficacy. In terms of the expected efficacy when both doses are given, ${\bar{\eta }}_{i}=\mu \eta_{i}$, where *μ* is the parameter that modulates the drop in efficacy; second, a number of eligible individuals may decide not to get vaccinated. This portion of the population is denoted as *α*. Therefore, the rate of change of individuals who take both doses of the vaccine (or the single-dose vaccine) is represented by the amount *τ*_*i*_*η*_*i*_ (1 − *α*)*V*_*i*_ (*t*), whereas for those who take only the first dose (when two are foreseen), or choose not to get vaccinated, it is expressed by $\tau_{i}{\bar{\eta }}_{i}\alpha V_{i}(t)$. The susceptible class is also fed back proportionally to the value of *α*. The general description of the model is provided in equation (2.1). The schematic representation of the model is shown in the electronic supplementary material. The conceptual definition of model parameters is shown in table 1.

2.1 $$\begin{array}{rl} & \frac{dS(t)}{dt}=-\beta (t)\frac{S(t)\;I(t)}{N}-\sum_{i=1}^{n}(\nu_{i}S(t)\\ & \;-\tau_{i}((1-\eta_{i})\;(1-\alpha )+(1-{\bar{\eta }}_{i})\alpha )\;V_{i}(t)),\\ & \frac{dI(t)}{dt}=\beta (t)\frac{S(t)I(t)}{N}-\left(\gamma +\rho +\sum_{i=1}^{n}\nu_{i}\right)I(t),\\ & \frac{dV_{i}(t)}{dt}=\nu_{i}(S(t)+I(t))-\tau_{i}V_{i}(t),\\ & \frac{dR(t)}{dt}=\gamma I(t)+\sum_{i=1}^{n}\tau_{i}(\eta_{i}(1-\alpha )+{\bar{\eta }}_{i}\alpha )\;V_{i}(t)\\ \mathrm{and}\; & \frac{dD(t)}{dt}=\rho I(t). \end{array}\}$$

Additionally, we employ the next-generation matrix method [26,27] to derive the effective reproduction number expression, which is given by

2.2 $$R(t)=\frac{\beta (t)S(t)}{N\left(\gamma +\rho +\sum_{i=1}^{n}\nu_{i}\right)}.$$

For detailed derivation, refer to the electronic supplementary material.

**Table 1.** Conceptual definition of model parameters. Association between symbols and their respective definitions, followed by measurement units.

symbol	definition (unit)
*β*	transmission rate (per day)
*ρ*	death rate (per day)
*γ*	removal rate (per day)
*ν*	vaccination rate (% of the population per day)
1/*τ*	interval between doses (day)
*η*	overall vaccine efficacy (—)
$\bar{\eta }$	overall impaired efficacy (—)
*α*	portion of people who have not received the second dose (—)

### 2.2. Case incidence and vaccination data

Daily data on confirmed cases and dead individuals due to COVID-19 in Rio de Janeiro are divided into two subsets, from before and during vaccination. We call training data both time series of infected and dead individuals in the interval between 10 March 2020, the first day with at least five cases diagnosed, and 19 January 2021, the last day before the start of vaccination. These data subsets are denoted as $D^{1}$ and $D^{2}$, respectively. In the other subset, which we refer to as test data, the time series are in the interval between 20 January, the day the vaccination started, and 27 October 2021. Cumulative data on individuals vaccinated with the first dose and immunized (with both doses or with the single-dose vaccine) are also adopted from the same period. There is no distinction regarding the type of vaccine in the available data. All data are obtained from the same public repository [28], which compiles the data provided by the Ministry of Health [29]. In turn, data on the distribution of vaccines for each Brazilian state, with the distinction among the types of vaccines, are obtained directly from the Ministry of Health website.

### 2.3. Data regularization

Daily data on infected and dead individuals in Rio de Janeiro are very noisy (see the electronic supplementary material, figure S2). Libotte *et al.* [30] analysed some of the reasons for this behaviour. The accumulation of confirmed cases that are not reported on weekends, in addition to a large-scale underreporting of cases and a reduced testing capacity are some of the main causes of such noise. The authors provide a numerical analysis of how data regularization using Gaussian Process Regression (GPR) can help reduce the effect of noise on parameter estimation. The study shows that when successive parameter estimates are performed, gradually adding data to the training set and comparing the corresponding model outcomes to the test set, there is great variability in the results. Such variability becomes evident when very different parameter estimates are obtained using slightly distinct training datasets (sometimes just adding one more datum to the training set). However, when the training set is regularized, the variability of the results has a remarkable reduction. In these circumstances, the regularization of data emerges as an alternative to reduce the noise level, without misrepresenting data behaviour, in order to streamline the task of fitting model responses to the dataset. This is our motivation for using regularized data in this study.

In particular, Gaussian Process (GP) models are a probabilistic approach to representing arbitrary functions by means of a probability distribution over all possible functions that fit a set of points [31]. GPR differs from regular regression models in that distributions are defined over functions, rather than their parameters, not requiring the definition of a parametric model that would be able to fit a set of observable data. The strength of GPs in steering experiments is due to the fact that realizations correspond to random functions, such that priors for unknown regression functions are provided and updated with knowledge of observable data. GPs depend on defining covariance functions (also known as kernels) that are used to define a similarity measure of the inputs [32]. Thus GPRs are able to avoid simple parametric assumptions (because it is a non-parametric approach), while providing uncertainty quantification on the predictions [33].

More formally, let $t={\left(t_{1},\ldots ,t_{\;p}\right)}^{⊤}$ denote the time training points associated to a set of *p*-dimensional observations $D={\left(D_{1},\ldots ,D_{\;p}\right)}^{⊤}$. Recalling the regular regression problem, $D_{i}=f(t_{i})+\varepsilon$, the function f : R→R maps a time training point into the data space (this is the GP we further expect to obtain), and $\epsilon ∼N(0,{\bar{\sigma }}^{2})$ is an additive independent and identically distributed Gaussian noise, where ${\bar{\sigma }}^{2}$ is the noise variance. Assuming that *t*, *t*^′^ ∈ **t** are a pair of general input vectors, a process given by *f*(*t*), defined according to its mean m(t)=E[f(t)] and a positive semi-definite kernel function $k(t,t^{\prime })=E[(f(t)-m(t))(f(t^{\prime })-m(t^{\prime }))]$, is said to be a GP represented by

$$f(t)∼GP\;(m(t),\;k(t,t^{\prime })).$$

The mean is often assumed to be zero (since the observed outputs can always be centred in order to have a zero mean).

In a regression problem, the prior probability density of $f(t)={\left(f(t_{1}),\ldots ,f(t_{\;p})\right)}^{⊤}$ has joint multivariate Gaussian distribution f∼N(0,K(t,t,λ)), such that K(t,t,λ) is the covariance matrix (which is also noise-dependent), whose entries are ${\left(K(t,t,\lambda )\right)}_{ij}=k(t_{i},t_{\;j},\lambda )+{\bar{\sigma }}^{2}\delta_{ij}$, for *i*, *j* = 1, …, *p*, where λ is the set of kernel hyper-parameters and *δ*_*ij*_ is the Kronecker delta. Now consider new input time points, denoted by t*, and their associated output values $D^{*}$, which we assume to be also normally distributed. The joint Gaussian distribution considering such points is given by

$$\left[\begin{array}{c} D\\ D^{*} \end{array}\right]∼N\left(0,\;\left[\begin{array}{cc} K(t,t,\lambda )+{\bar{\sigma }}^{2}I & K(t,t^{*},\;\lambda )\\ K(t^{*},t,\lambda ) & K(t^{*},t^{*},\lambda ) \end{array}\right]\right),$$

where **I** is the *p* × *p* identity matrix. Therefore, by deriving the conditional distribution [31], the posterior predictive equation is the multivariate Gaussian distribution

$$p(D^{*}|t^{*},t,D)=N(\mu^{*},\Sigma^{*}),$$

with mean

$$\mu^{*}=K(t^{*},t,\lambda ){\left(K(t,\;t,\lambda )+{\bar{\sigma }}^{2}I\right)}^{-1}D$$

and covariance matrix

$$\Sigma^{*}=K(t^{*},t^{*},\lambda )-K(t^{*},t,\lambda )(K(t,t,\lambda )+{\bar{\sigma }}^{2}I{)}^{-1}K(t,\;t^{*},\lambda ).$$

As new pairs $\left(t^{*},D^{*}\right)$ are incorporated into the regression problem, the mean set $\mu^{*}$ is updated and adopted as the output of the GPR model, whereas $\Sigma^{*}$ provides a measure of confidence in the estimate [34]. In this work, we adopt the RBF (radial basis function) kernel [31],

$$k(t,t^{\prime },\ell )=\exp\left(-\frac{|t-t^{\prime }{|}^{2}}{2\ell^{2}}\right),$$

where λ=(ℓ) is the length scale of the kernel. For the available training data, the optimal hyper-parameter values are ℓ=51.1 days and ℓ=29.1 days for the daily data of infected $\left(D^{1}\right)$ and dead $\left(D^{2}\right)$ individuals, respectively. Regularized data are shown in the electronic supplementary material, figure S2.

### 2.4. Inference of model parameters

Model outcomes are fitted to the training set using Bayesian inference. As the training data are from the period prior to the start of vaccination, all model parameters that are associated with vaccination (*α*, *ν*_*i*_, *τ*_*i*_, *η*_*i*_ and ${\bar{\eta }}_{i}$, for *i* = 1, …, *n*) are set to zero at this point. In this setting, the model of equation (2.1) reduces to the SIR model (including the dead class). As for the remaining parameters, we take the removal rate and the death rate as biological parameters. The removal rate is equal to *γ* = 0.06 [35] and the mortality rate is inferred considering the number of cases and deaths, on average, since the day the first case was reported. Since 5 March 2021, when the first case was confirmed in the state of Rio de Janeiro, 607 days have passed, and 2174 cases and 113 deaths, on average, per day have been reported. Thus, we infer the mortality rate as ρ = 113/2174 = 0.05197 per day.

Regarding the transmission rate, we adopt the functional form given by

2.3 $$\beta (t)=\beta_{1}\exp(-\beta_{2}t)+\beta_{3}\exp(\beta_{4}t).$$

This specific choice is motivated by the fact that, in the particular time period over which the training data span, there seems to be the incidence of two waves of infection. The contribution of the term associated with the negative exponential would be able to represent the infection rate at an early stage when few individuals are immune, and the contact rate between them leads to an increase in the incidence of cases until the peak of the first wave is reached. On the other hand, the contribution of the term associated with the positive exponential would be related to a new increase in the infection rate after the event of the first wave. Therefore, the parameters to be estimated are $\theta =(\beta_{1},\beta_{2},\beta_{3},\beta_{4})$.

To update the Bayesian model, we employ the Transitional Markov Chain Monte Carlo [36] (TMCMC) method. This sequential particle filter method combines aspects of simulated annealing optimization [37] with Markov Chain Monte Carlo sampling. To infer the parameters θ, we initially obtain the set of estimators that generate the model outcomes that best fit the regularized training data, through least squares. Such values are denoted by ${\hat{\beta }}_{1},\ldots ,{\hat{\beta }}_{4}$ and, in turn, are used to define the prior distribution p(θ) of the corresponding parameters (our prior belief about the distribution of θ), which we assume to be uniformly distributed,

2.4 $$p(\beta_{\;j})∼U({\hat{\beta }}_{\;j}(1-\xi ),{\hat{\beta }}_{\;j}(1+\xi )),$$

where 0 < *ξ* < 1 is a relative displacement. In this particular application, the prior distribution of *β*_*j*_ is defined symmetrically around ${\hat{\beta }}_{\;j}$, for j∈{1,2,3,4}, with *ξ* = 0.9. This strategy aims to bypass parameter identification problems [38].

The likelihood p(D|θ) expresses the plausibility of observing the data, given a specific θ. In this work, we assume that the likelihood follows a normal distribution

2.5 $$p(D^{q}|\theta )∼N(\mathrm{mean}=M^{q},\;\mathrm{variance}=\sigma_{q}^{2}).$$

Correspondingly to the data, the model responses $M^{q}$ represent the number of infected and dead individuals in equation (2.1), for q∈{1,2}, respectively. Of note, the model measures the cumulative number of dead individuals. Therefore, it is mandatory to differentiate the result obtained by the numerical approximation of equation (2.1), regarding compartment *D*, so that $M^{2}$ is consistent with $D^{2}$. As the variance of the distribution is not known, it plays the role of a hyper-parameter and must therefore be estimated together with θ. In the sampling process, the solution of the system given by equation (2.1) is approximated using the Fehlberg method [39]. For this purpose, the initial conditions adopted are $I(0)=D_{1}^{1}$, $D(0)=D_{1}^{2}$, *R*(0) = 0 and, therefore, *S*(0) = *N* − *I*(0) − *D*(0) − *R*(0).

Since TMCMC gradually pushes the samples from the prior distribution to the posterior target distribution, the samples of the intermediate distributions are used to obtain an estimate of the evidence p(D). Therefore, the information from equations (2.4) and (2.5) is combined to compose the posterior distribution of the parameters,

$$p(\theta |D^{q})\propto p(D^{q}|\theta )p(\theta ).$$

Information from observable data is employed to update the prior belief about the model’s parameters to a posterior belief, simultaneously considering data from infected and dead individuals. Posterior distributions are approximated using 2000 samples. To compute the 95% credible intervals (whose values are shown in parentheses following each numerical result in this work), we adopt an equal-tailed interval, by computing the 2.5th and 97.5th percentiles of the posterior distribution p(θ|D); that is, 2.5% of the distribution on either side of its limits. In turn, the maximum a posterior (MAP) of each parameter is approximated by computing the maximum value of the probability density function estimated using the kernel density estimator (KDE) [40,41].

## 3. Results

**Figure 1.**
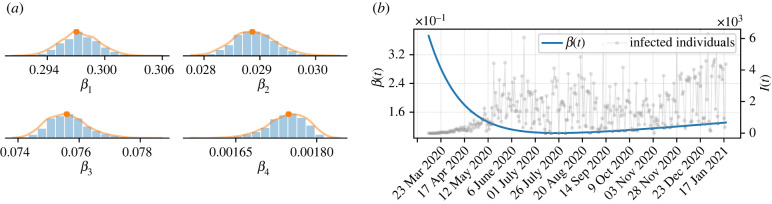
Optimal parameters and transmission rate. (*a*) Histograms of the posterior probability distribution of independent *β*(*t*) parameters. The orange lines represent the approximations of the probability distributions computed using the KDE and the orange dots are the MAP estimates of each parameter. (*b*) Time-dependent transmission rate, simulated with the MAP of the inferred parameters.

To perform the simulations, it is first necessary to infer the values of the free parameters of the model, whose model outcomes best fit the regularized training data. Figure 1*a* shows the posterior distributions of parameters *β*_1_, …, *β*_4_, whose statistics are detailed in the electronic supplementary material, table S3. The solid orange curves represent the approximation of the distribution computed by KDE. In turn, the orange dots are the MAPs of each parameter. Figure 1*b* shows the behaviour of the function that describes the transmission rate, given by equation (2.3), using the MAP values, for the time period over which the training data span. In the early stage of the outbreak, with more frequent contact between people and in the absence of pharmaceutical interventions, the transmission rate was at a high level, gradually decreasing during the first wave of infections, approximately until the end of July 2020. A further increase in the transmission rate led to the second wave, which remained at a high level of transmission for months until its growth could be halted by the start of vaccination.

**Figure 2.**
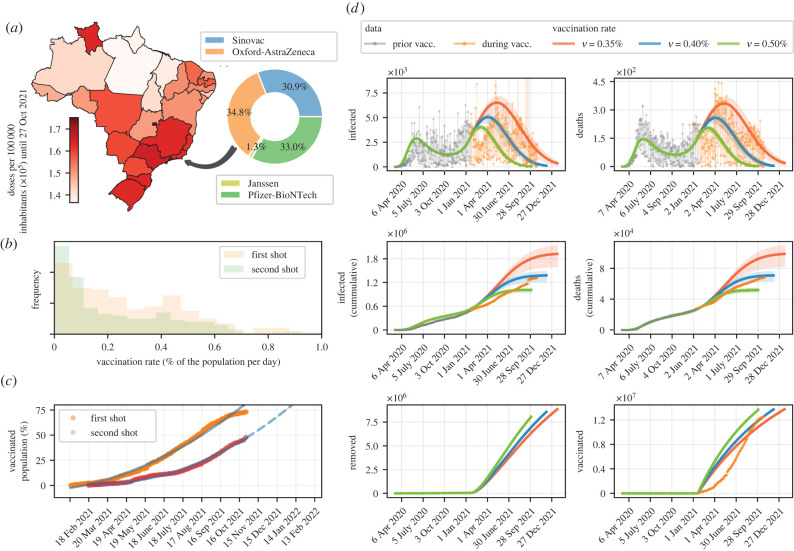
Speed of vaccination and expectation of disease mitigation in Rio de Janeiro. (*a*) Map of vaccine doses distributed in each Brazilian state per 100 000 inhabitants, and the proportion of each type of vaccine destined for Rio de Janeiro, until 27 October 2021. (*b*) Frequency of vaccination rate for each shot, in terms of percentage of population per day. (*c*) Percentage of coverage of the population eligible for vaccination for each shot. (*d*) Model simulation considering different vaccination rates, slow (ν=0.35%), intermediate (ν=0.40%) and fast (ν=0.50%), taking into account the frequencies (*b*) and the proportion of vaccines of each type (*a*). The model is simulated until reaching the same amount of vaccines administered in each scenario (see the lower right frame). The solid lines represent the simulations using the MAP values and the shaded areas represent the 95% credible intervals. Note that the period of the end of vaccination in the scenario where an intermediate vaccination rate is adopted agrees with the prognosis in (*c*).

We consider that the target of individuals to be immunized in Rio de Janeiro is proportional to 80%, which also corresponds to the number of inhabitants aged 12 years or over [42]. The vaccination process is carried out with four vaccines (*n* = 4), and the vaccination rates of each one are proportional to the number of doses granted to Rio de Janeiro by the Ministry of Health, as shown in figure 2*a*. Table 2 shows the attributes related to each vaccine’s efficacy and dosage (including the interval between doses) adopted in this study. The simulations are conducted considering three scenarios related to the overall vaccination rate: the base scenario is associated with the average vaccination rate at the time of writing this paper; that is, ν=0.40% of the population vaccinated per day. This corresponds to approximately 69 200 vaccinated individuals per day, which agrees with the average of daily vaccinations. In the two other hypothetical scenarios, we consider ν=0.35% and ν=0.50% of the population vaccinated per day. In this setting, approximately 60 550 and 86 500 individuals are vaccinated per day, on average, respectively. Taking into account the situation of asymptomatic individuals and a poor testing policy, which leads to substantial underreporting of cases, we adopt the same vaccination rate for susceptible and infected individuals [48,49]. Figure 2*b* shows the frequencies of vaccination rates considering both shots (single-dose vaccines count as second doses), given the cumulative number of individuals vaccinated per day, which in turn is shown in figure 2*c*. The target vaccination coverage for the first dose would be reached in approximately 290 days, which means that 80% of the population would have received at least the first dose by November 2021, as supported by the prediction shown in figure 2*c*. As for the second dose, the prediction indicates that the population would be immunized in the first months of 2022, respecting the interval between doses.

**Table 2.** Characteristics of the vaccines used in the simulations, in terms of overall efficacy and interval between doses (when applicable). Of note, the recommended inter-dose interval for Pfizer-BioNTech vaccines is 21–28 days [43]. However, for countries that face a high incidence of COVID-19 cases and that have not yet achieved safe vaccination coverage rates, the World Health Organization recommends that the interval between doses be extended to 12 weeks [44], which has been adopted all states of Brazil.

vaccine	efficacy	dosage	source
Janssen	66.9%	single-dose	[45]
Oxford-AstraZeneca	76%	two doses, 12 weeks apart	[46]
Pfizer-BioNTech	95%	two doses, 12 weeks apart	[43]
Sinovac	50.34%	two doses, 4 weeks apart	[47]

### 3.1. Benefits and risks regarding the pace of vaccination

The influence of the pace of vaccination on the mitigation of the epidemic, in the matter of reducing the number of infected and dead individuals over time, is shown in figure 2*d*. Note that the vaccination data agree with the simulations, even if they were not used to estimate the model parameters (only $D^{1}$ and $D^{2}$ were used in the parameter estimation). The same goes for the cumulative data from infected and dead individuals, indicating that the choice of model parameters seems to correspond to the actual epidemic scenario in Rio de Janeiro. Such simulations indicate that if the vaccination process were faster, allowing the vaccination of approximately 8650 more people per day (with ν=0.5%) compared to the amount vaccinated in the base scenario, on average, the number of cases could be reduced by 26.58%, from 1 378 382 (1 226 446–1 472 044) to 1 011 948 (944 239–1 054 368) cases, whereas the death toll would drop from 70 846 (63 037–75 659) to 52 013 (48 532–54 193). On the other hand, when the pace of vaccination is delayed proportionally to ν=0.35%, the adverse effect is disproportionately greater: the number of confirmed cases would rise to 1 922 585 (1 607 030–2 128 694), a meaningful increase of 39.48%, and deaths could reach 98 812 (82 596–109 401).

### 3.2. How the timing of vaccination roll-out affects disease mitigation

**Figure 3.**
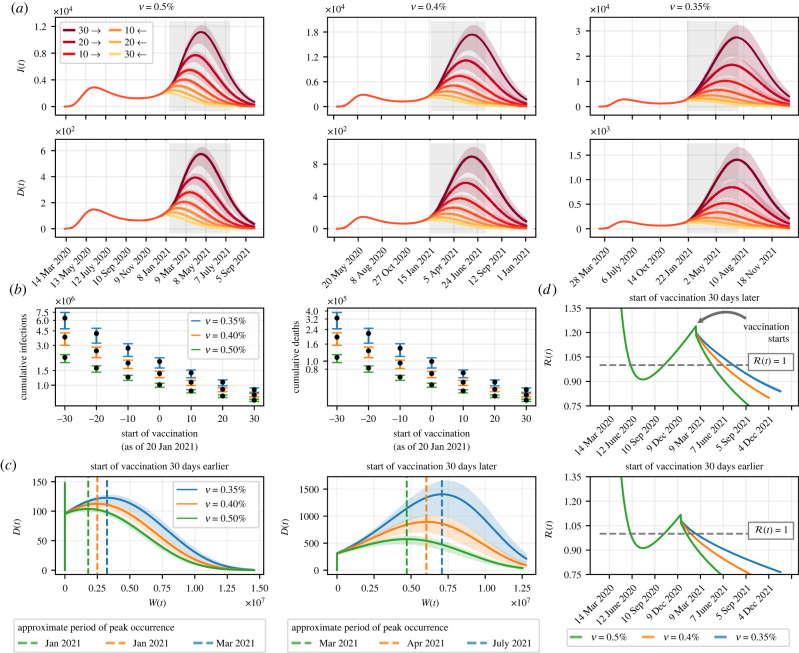
Importance of rolling out the vaccination programme as soon as possible. (*a*) Simulations considering scenarios in which the start of vaccination is advanced or delayed by up to 30 days in relation to the actual start date. The grey shaded area represents the six-month interval from the start of vaccination, 20 January 2021. (*b*) Cumulative number of dead and infected individuals when reaching 80% vaccination coverage, varying the day on which vaccination is started, as well as vaccination rates. The error bars are associated with the 95% credible interval of the simulations and the black dots refer to the simulations when the maximum a posterior of the inferred parameters are adopted. (*c*) Relationship between the number of individuals vaccinated and dead over time, given a 30-day early or late start in vaccination in relation to the actual date. The vertical dashed lines express the approximate period at which deaths would peak, for each particular vaccination rate. (*d*) Effective reproduction number, given the analysed scenarios. The solid lines represent the simulation using the MAP value and the shaded areas represent the 95% credible interval.

Here, we propose hypothetical scenarios in which the vaccination efforts get underway 10, 20 or 30 days before or after 20 January 2021. For each particular vaccination rate, we simulate the model for all combinations of proposed scenarios, whose outcomes are shown in figure 3*a*, concerning the daily number of infected and dead individuals. For an arbitrary vaccination rate, it is clear that starting the vaccination campaign a few days earlier is beneficial both in terms of ‘flattening the curves’ and in terms of suppressing the epidemic. Take as an example the scenario in which ν=0.40%. On 4 June 2021, when simulations show that the daily death toll would peak if vaccination had started 30 days late, there would have been 893 deaths (700–1001). On the same day, had the start of vaccination been 30 days early, there could have been only 34 deaths (28–38). Note that in the latter case, deaths would peak on 5 June 2020 (first wave), at 148 deaths (146–151).

Figure 3*b* shows how delaying the start of vaccination combined with vaccination at a slow pace could be devastating to the population. In the worst-case scenario, with vaccination coverage increasing slowly (ν=0.35%) and the vaccination campaign starting 30 days after 20 January 2021, the number of infected individuals could have reached 6 398 467 (4 754 038–7 462 813), whereas there could have been 328 781 deaths (244 308–383 445). If we look at the opposite scenario, when more effort is put into a rapid vaccination (ν=0.50%) that started 30 days before the actual day, the number of cases and deaths would drop to 660 212 (637 960–677 854) and 33 935 (32 791–34 841), respectively.

We also sought to directly relate the number of vaccinated and dead individuals, aiming to analyse the likely hardship to the population when the start of vaccination is delayed, compared to the scenario in which vaccination had started earlier. Suppose vaccination had started on 19 February 2021, 30 days beyond the actual date, when 313 deaths (291–324) would have been confirmed, as shown in figure 3*c*. Based on the benchmark vaccination rate, the simulations show that the deaths would peak approximately in June 2021, at 893 deaths (700–1001). At this time, about 6 028 386 people (6 026 531–6 032 208) could have been vaccinated (with both doses and with the single-dose vaccine), representing approximately 34.84% of the population. However, the worst-case scenario would bring out a far more ruthless possibility: even with approximately 7 081 206 people immunized (7 078 053–7 091 406), deaths would peak in July 2021, reaching 1405 deaths in a single day (1010–1640). This means that, despite having vaccinated nearly 17.46% more people, comparing both scenarios, a record-high daily death toll could have been reached, to a great extent driven by the late start of vaccination.

According to the simulations, in 2020 the effective reproduction number (see the electronic supplementary material) was only below the threshold R(t)=1 between June and October, as shown in figure 3*d*. Despite that, in this period the lowest value reached was R(t)=0.912, at the end of July. Afterwards, the effective reproduction number was always above one, until the vaccination started to take effect. At this point, imagine that the vaccination had been brought forward by 30 days. On the same day as the start of vaccination, 1834 new cases (1786–1862) would have been confirmed. Even maintaining a slow pace of immunization (ν=0.35%), the transmission potential of SARS-CoV-2 could have reached R(t)=1 as early as February 2021 (approximately three months after the hypothetical start of vaccination), when Rio de Janeiro would have vaccinated 24.19% of the eligible portion of the population.

### 3.3. Potential aftermath of COVID-19 vaccine hesitancy

**Figure 4.**
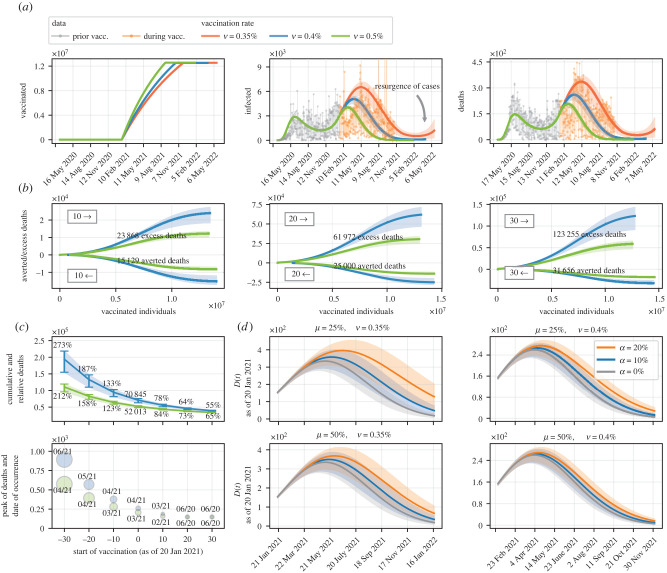
Flawed vaccination policy and excess deaths. (*a*) Model simulation where part of the population eligible to be vaccinated does not receive any dose. (*b*) Ratio between the number of deaths given potential scenarios in which the start of vaccination is ahead of the actual date. Scenarios where vaccination would be implemented 10, 20 and 30 days before 20 January 2021 are considered, as well as two vaccination rates (ν=0.40% and ν=0.50%), and excess deaths are estimated. (*c*) Variation in the cumulative number of deaths and the number of deaths at the peak of the epidemic curve (during vaccination) taking into account the start of vaccination on different days. The relative percentage amount of cumulative deaths is shown, as well as the month in which deaths would peak. (*d*) Simulation considering that part of the population proportional to *α* does not take the second dose of the vaccine. Two scenarios are considered in which only the first dose of the vaccine has efficacy equivalent to *μη*, combined with two vaccination rates (ν=0.35% and ν=0.40%). The solid line represents the simulation using the MAP value and the shaded areas represent the 95% credible interval.

We now simulate the model considering only 70% vaccination coverage, a reduced amount due to people who are unwilling to be vaccinated. Figure 4*a* shows the model outcomes for the daily number of infected and dead individuals over time, given the three vaccination rates we have assumed, alongside the cumulative number of vaccinations. Considering the benchmark vaccination rate, 70% vaccination coverage would be reached in the first half of October 2021, when simulations indicate that 485 cases (290–629) would be reported daily. In the same period, but assuming a slower vaccination rate, the daily number of confirmed cases would be 2171 (1280–2842), when 64.09% of the population would be immunized. In addition, overall low vaccination coverage combined with a lethargic immunization program could raise the possibility of a resurgence of cases (and hence deaths) as early as 2022. After experiencing a reduction in the number of cases, to a large extent due to vaccination, in February 2022 there would be the smallest number of infected individuals since the onset of the epidemic, 519 (206–856). However, in the following months, the incidence of cases could increase again, reaching 1188 new cases (313–2492) per day by mid-May 2022.

Thousands of people have also been missing their second dose of vaccine in Rio de Janeiro, further complicating a campaign already marred by backwardness and supply shortages. To the best of our knowledge, there are still no studies that confirm the overall efficacy of all vaccines used in Rio de Janeiro when only one shot is provided (except for the Janssen vaccine), although some studies have already reported relevant results [50–54]. In the absence of such information, we assume two scenarios regarding vaccine efficacies when only the first shot is given: that is, efficacies are weakened proportionally to μ=25% and μ=50%. Surveys show that around 14.5% of the Brazilian population somewhat disagree, strongly disagree, or remain neutral regarding vaccination [18]. Within this context, we also consider scenarios with low (α=20%) and moderate (α=10%) demand for the second dose of vaccines (when applicable), as well as the best scenario in which α=0%. Simulations for the number of dead individuals as of the actual day when vaccination started, combining factors associated with parameters *μ* and *α*, are shown in figure 4*d*. Initially, assume that the first dose of vaccines would yield an efficacy proportional to μ=25% of the overall efficacy when both doses are given. In a scenario subject to slow vaccination, 70% of the eligible population would have been immunized in approximately 211 days. After this time frame, the number of daily deaths would be 357 (236–437) if 20% of the population eligible to be vaccinated missed their second dose. If the percentage of individuals who do not receive the second dose dropped to 10%, the death toll would be 263 (174–322) in the same period. In turn, if the efficacy of vaccines were weakened by μ=50% when the second dose is neglected, the number of dead individuals on the same day could be 292 (193–358) and 237 (156–290), bearing in mind the two scenarios related to vaccination coverage with the second dose, respectively.

### 3.4. More ambitious vaccination targets and avertable deaths

We attempt to infer deaths that could have been averted simply by having vaccination started days earlier or if the daily rate of vaccination had been higher. Figure 4*b* shows the relationship between vaccinated individuals and cumulative deaths over time. We simulate the model using the benchmark vaccination rate (ν=0.40%) and compare the outcomes in the context of a faster vaccination (ν=0.50%), making allowance for different days for the start of vaccination from the day it actually started. Simulations show that presumably not-so-challenging measures, such as having anticipated the vaccination campaign roll-out by just ten days, combined with an average vaccination rate approximately 25% faster, could have averted 15 129 deaths (12 029–16 986) in relation to the actual scenario; from a more optimistic, yet still realistic, perspective on the vaccination roll-out, consider a 30-day advance on the date on which the campaign actually started. In this framework, 31 657 deaths (25 801–35 117) could have been prevented, which represents 44.68% of the deaths (40.93–46.41%) that would have occurred since vaccination was started, assuming a vaccination rate equal to ν=0.40%.

When vaccination became available in Rio de Janeiro, 490 821 cases and 28 215 deaths had already been reported (on that day, there were 4015 new cases, with 189 deaths). Figure 4*c* shows that by increasing the vaccination rate, and even under the hypothesis of delay, the number of deaths could drop to 110 653 (95 920–119 456) when the target vaccination coverage in October 2021 had been reached. By contrast, starting mass vaccination 30 days before 20 January 2021, when Rio de Janeiro had 457 160 cases (448 776–467 785) and 23 546 deaths (23 114–24 093), could have caused the number of deaths to drop to 20% compared to the worst-case scenario, assuming that ν=0.40%.

Such delays can also further increase the incidence of the disease, delaying its peak and, consequently, causing the peak of deaths to be shifted forward. According to simulations, at the worst stage of the epidemic, there could be up to 893 deaths (706–1003) a day if there had been a 30-day delay in making vaccines available to the population, considering the benchmark vaccination rate, shifting the peak two months ahead of what is expected without such a delay. Figure 4*c* supports the fact that delays in the start of the vaccination campaign cause adverse effects that are more severe when the vaccination process is slower.

## 4. Discussion

Until 27 October 2021, approximately 290 days since the roll-out of vaccination in Brazil, Rio de Janeiro was one of the states that had received the most doses per 100 000 inhabitants, about 163 561, as shown in figure 2*a*. Altogether, Brazil had about 155 727 doses per 100 000 inhabitants. In general, access to vaccines in Brazil was delayed, and this ends up affecting the pace of vaccination even in the states with the most supply of doses. This fact becomes clear when we place the situation in Brazil side by side with that of some other countries, such as Canada, in terms of access to vaccines. Vaccination was launched in Canada on 14 December 2020, nearly one month earlier than in Brazil. In the 290-day window since the launch of the vaccination in Canada, approximately 147 820 doses per 100 000 inhabitants had been administered, a pace similar to what had been performed in Brazil. However, as of 27 October 2021, Canada had reached around 153 480 vaccines administered per 100 000 inhabitants. Considering a 7-day rolling average of daily new deaths, at that time Canada had 0.89 deaths per million people, whereas Brazil had 1.64 deaths [55]. Such statistics shed light on the importance of getting vaccinated as soon as possible.

Social mobility and NPIs are also important factors when analysing the course of vaccination. The engagement of the Brazilian population in such measures has always been below expectations [56]. A very relevant fact is that only 45.5% of Brazilians say they wear a face mask outside the home [57]. Our findings show that the possibility of an eventual resurgence of cases in 2022 should not be overlooked, even though most of the population has been vaccinated. This concern even brings up discussions about the possible loss of immunity and the need for extra doses [58], although vaccines may remain limited, especially in low-income countries [59], making NPIs essential even after achieving adequate vaccine coverage.

In this study, we simplify the effect of vaccines to provide ‘instant immunity’. In the proposed model, this follows from the fact that the rate at which vaccinated individuals move into the removed class is given by *τ*_*i*_*V*_*i*_(*t*); that is, individuals become immunized instantly after the interval between doses. In fact, immunity only arises a few days after the second dose (when applicable). However, the model is simulated for several days longer than the time required for immunity to be achieved—usually around two weeks. Therefore, we assume that our results would not be greatly influenced by this latent period, especially as the number of vaccinated individuals would not change. On the other hand, such simplification of the model reduces the number of parameters to be analysed, so that greater focus is given to parameters and quantities related to the objectives of the work. This motivates us to consider the choice of not including a delay in immunization after the second dose (or after receiving the single-dose vaccine).

As for the values of vaccine efficacies, it is important to emphasize that they refer to the probability of preventing severe disease and death. However, we assume that vaccinated individuals can still be infected. This hypothesis is in line with the expected effect of vaccines [43,45,47]. In the proposed model, infected individuals, even having been vaccinated, can die. The frequency of deaths is preeminently related to the efficacy of the vaccine. However, note that in our model, vaccine efficacy has no relevant effect on transmission rate, but on the rate at which individuals are moved into the removed compartment. Therefore, the vaccination rate acts in the system of differential equations as a modulator of the rate at which individuals are moved from the infected to the dead compartment, as vaccines prevent severe disease and therefore reduce the mortality rate, but with no significant effect on the transmission rate [60].

The analysed scenarios reflect current knowledge about vaccination in Rio de Janeiro, from the perspective of available data. The persistence of such predictions depends to some extent on the confirmation of the hypotheses put forward. Particularly regarding vaccine hesitancy (whether for both doses or just the second), the inaction of certain people depends a lot on facts that cannot be predicted. Despite this, social network posts can provide insight into attitudes and sentiments towards vaccination, for instance. From 1 December 2020 to 31 March 2021, a lexicon-based sentiment analysis of Twitter posts shows a steady trend in people’s perception of Pfizer and Moderna vaccines, while hesitation over the Oxford-AstraZeneca vaccine appears to be increasing over time [61]. Nevertheless, until 27 October 2021, Oxford-Astrazeneca vaccines supplied most of the Brazilian demand, with 34.8% of all vaccines distributed so far (figure 2*a*). This could be an indication that, even if the vaccine is available, popular sentiment may be volatile enough that eventually people would not return for the second dose or possible additional doses, especially for vaccines where the interval between doses is high. In this context, social networks play a fundamental role in shaping the opinion of part of the population, since the sharing of narratives and personal opinions without scientific background comes to the knowledge of many people [62,63]. In Brazil, the oscillations regarding the feelings analysed in the posts on social networks are due, in large part, to political actions [64].

## Data Availability

Code to replicate analysis and figures supporting the findings of the manuscript is available via the project GitHub repository at https://github.com/gustavolibotte/vaccines-COVID-19. The code is licensed under the MIT license. Source data are provided in this paper and all data used in this study can be downloaded from the cited sources. The data are provided in electronic supplementary material [65].
